# Host contact dynamics shapes richness and dominance of pathogen strains

**DOI:** 10.1371/journal.pcbi.1006530

**Published:** 2019-05-21

**Authors:** Francesco Pinotti, Éric Fleury, Didier Guillemot, Pierre-Yves Böelle, Chiara Poletto

**Affiliations:** 1 INSERM, Sorbonne Université, Institut Pierre Louis d’Épidémiologie et de Santé Publique (IPLESP), 75012 Paris, France; 2 INRIA, Paris, France; 3 Inserm, UVSQ, Institut Pasteur, Université Paris-Saclay, Biostatistics, Biomathematics, Pharmacoepidemiology and Infectious Diseases (B2PHI), Paris, France; University of California, Los Angeles, UNITED STATES

## Abstract

The interaction among multiple microbial strains affects the spread of infectious diseases and the efficacy of interventions. Genomic tools have made it increasingly easy to observe pathogenic strains diversity, but the best interpretation of such diversity has remained difficult because of relationships with host and environmental factors. Here, we focus on host-to-host contact behavior and study how it changes populations of pathogens in a minimal model of multi-strain interaction. We simulated a population of identical strains competing by mutual exclusion and spreading on a dynamical network of hosts according to a stochastic susceptible-infectious-susceptible model. We computed ecological indicators of diversity and dominance in strain populations for a collection of networks illustrating various properties found in real-world examples. Heterogeneities in the number of contacts among hosts were found to reduce diversity and increase dominance by making the repartition of strains among infected hosts more uneven, while strong community structure among hosts increased strain diversity. We found that the introduction of strains associated with hosts entering and leaving the system led to the highest pathogenic richness at intermediate turnover levels. These results were finally illustrated using the spread of *Staphylococcus aureus* in a long-term health-care facility where close proximity interactions and strain carriage were collected simultaneously. We found that network structural and temporal properties could account for a large part of the variability observed in strain diversity. These results show how stochasticity and network structure affect the population ecology of pathogens and warn against interpreting observations as unambiguous evidence of epidemiological differences between strains.

## Author summary

Pathogens are structured in multiple strains that interact and co-circulate on the same host population. This ecological diversity affects, in many cases, the spread dynamics and the efficacy of vaccination and antibiotic treatment. Thus understanding its biological and host-behavioral drivers is crucial for outbreak assessment and for explaining trends of new-strain emergence. We used stochastic modeling and network theory to quantify the role of host contact behavior on strain richness and dominance. We systematically compared multi-strain spread on different network models displaying properties observed in real-world contact patterns. We then analyzed the real-case example of *Staphylococcus aureus* spread in a hospital, leveraging on a combined dataset of carriage and close proximity interactions. We found that contact dynamics has a profound impact on a strain population. Contact heterogeneity, for instance, reduces strain diversity by reducing the number of circulating strains and leading few strains to dominate over the others. These results have important implications in disease ecology and in the epidemiological interpretation of biological data.

## Introduction

Interactions between strains of the same pathogen play a central role in how they spread in host populations. [1–7]. In *Streptococcus pneumoniae* and *Staphylococcus aureus*, for instance, several dozen strains can be characterized for which differences in transmissibility, virulence and duration of colonization have been reported in some cases [8, 9]. Strain diversity may also affect the efficacy of prophylactic control measures such as vaccination or treatment. Indeed, strains may be associated with different antibiotic resistance profiles [3, 5, 10, 11], and developed vaccines may only target a subset of strains [2, 3, 12]. With the increasing availability of genotypic information, it has become easy to describe the ecology of population of pathogens and to monitor patterns of extinction and dominance of pathogen variants [13–17]. However, the reasons for multi-strain coexistence patterns (e.g. coexistence between resistant and sensitive strains) or dominance of certain strains (e.g. in response to the selection pressure induced by treatment and preventive measures) remain elusive. One may invoke selection due to different pathogen characteristics, but also environmental and host population characteristics, leading to differences in host behavior, settings and spatial structure may affect the ecology of strains [14–19]. In particular, human-to-human contacts play a central role in infectious disease transmission [20]. This is increasingly well described thanks to extensive high-resolution data—including mobility patterns [21–23], sexual encounters [24], close proximity interactions in schools [25, 26], workplaces [27], hospitals [16, 28–31], etc.—that enable basing epidemiological assessment on contact data with real-life complexity [32, 33]. For instance, the frequency of contacts can be highly heterogeneous leading more active individuals to be at once more vulnerable to infections and acting as super-spreaders after infection [24, 33–35]. Organizational structure of certain settings (school classes, hospital wards, etc.) and other spatial proximity constraints lead to the formation of communities that can delay epidemic spread [36, 37]. Individual turnover in the host population is also described as a key factor in controlling an epidemic [20, 38]. It is likely that, since they impact the spread of single pathogens, the same characteristics could affect the dynamics in multi-strain populations. It was shown, indeed, that network structure impacts transmission with two interacting strains [39–46], the evolution of epidemiological traits [47–49] and the effect of cross-immunity [50, 51]. Yet in these cases, complex biological mechanisms—such as mutation, variations in transmissibility and infectious period, cross immunity—were used to differentiate between pathogens, thereby making the role of network characteristics difficult to assess in its own right.

For this reason, we focused on the dynamical pattern of human contacts and examined whether it contributes to shaping the population ecology of interacting strains under minimal epidemiological assumptions regarding transmission. We described a neutral situation where all strains have the same epidemiological traits and compete via mutual exclusion (concurrent infection with multiple strains is assumed to be impossible) in a Susceptible-Infected-Susceptible (SIS) framework. We studied the spread of pathogens in a host population during a limited time window, disregarding long-term evolution dynamics of pathogens. More precisely, new strains were introduced through host turnover rather than *de novo* mutation or recombination in pathogens. We quantified the effect of network properties on the ecological diversity in strain populations with richness and dominance indicators. We assessed in turn heterogeneities in contact frequency, community structure and host turnover by comparing simulation results obtained with network models exhibiting a specific feature. We then interpreted *S. aureus* carriage in patients of a long-term care facility in the light of these results.

## Results

### Multi-strain spread on dynamical networks

We simulated the stochastic spread of multiple strains on a dynamical contact network of individuals (nodes of the network). Individuals can be either susceptible or infected with a single strain at a given time, and, for each strain, *β* and *μ* indicate the transmission and the recovery rate respectively. We assumed turnover of individuals, who enter the system with rate λ_in_, and associated injection of previously unseen strains, carried by incoming individuals with probability *p*_*s*_. We considered synthetic network models, each displaying a specific structural feature, as well as a real network reconstructed from close-proximity-interaction data collected in a hospital facility. We calibrated all network models to the same average quantities—average population size $\bar{V}$, fraction of active nodes $\bar{a}$, average degree $\bar{k}$ and strength of the community repartition *p*_*IN*_, when applicable—that were chosen to correspond with the hospital network used in the application. Epidemiological parameters were motivated by the duration of *S. aureus* carriage in patients. A larger range of values was explored in some cases to address their impact on the dynamics. We analyze the structure of strain population at the dynamic equilibrium by computing, for each network model, ecological diversity measures, including species richness and evenness/dominance indices [52, 53]. All details about network models, numerical simulations and ecological indicators are described in the Materials and methods section.

### Effects of contact heterogeneity

In order to probe the effect of contact heterogeneity on strain ecology we compared a homogeneous model (HOM) in which all nodes have the same activity potential, i.e. they have equal rate of activation to establish contacts, with a heterogeneous model (HET), akin to the activity-driven model described in [34], where the activity potential is different across nodes and is drawn from a power-law distribution.

**Fig 1:**
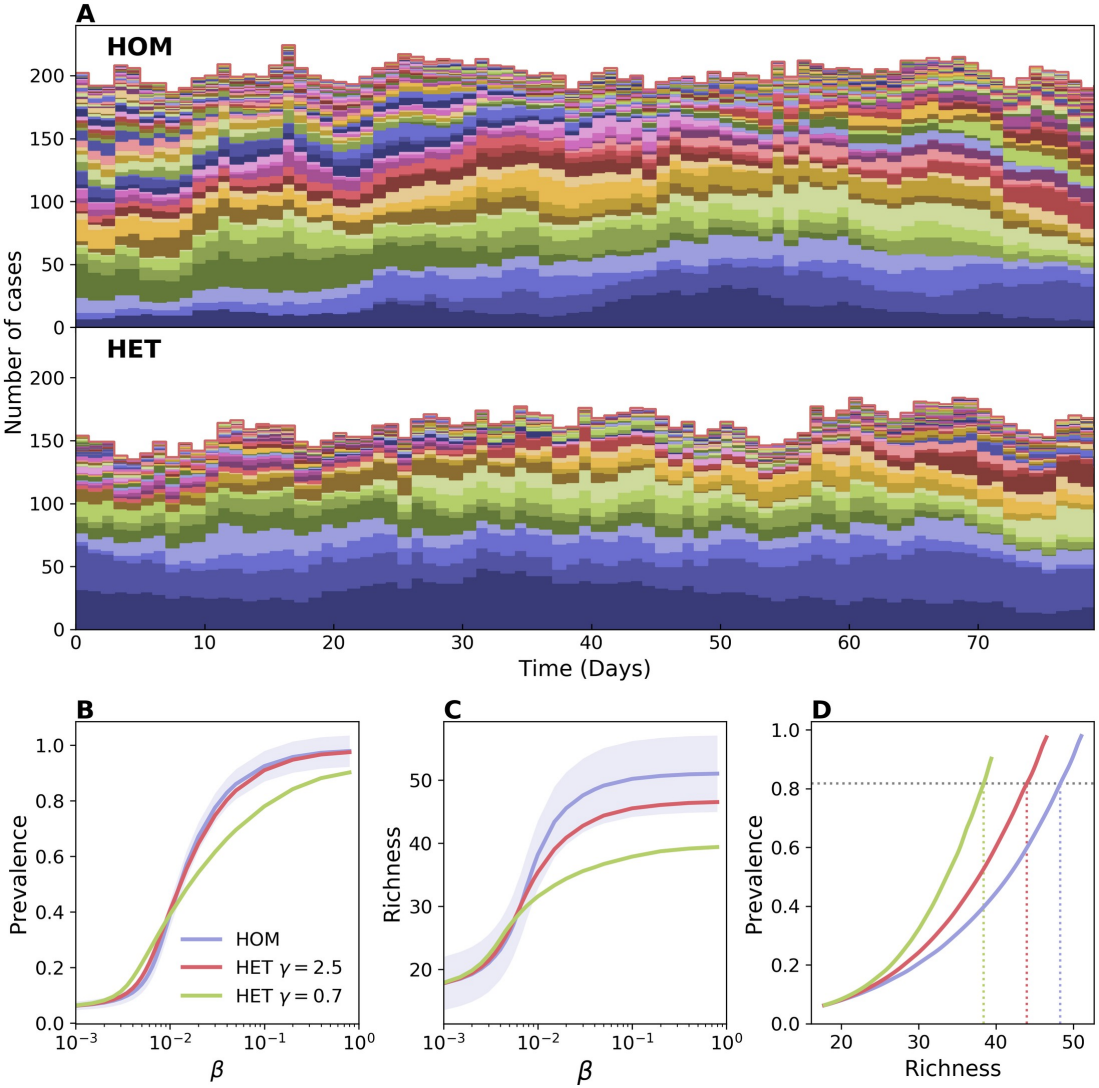
Effect of contact heterogeneity on strain richness. Comparison between a homogeneous (HOM) and a heterogeneous (HET) network. In HOM all nodes have the same activity rate *a*_H_ = 0.285 and the network average degree is $\bar{k}=0.89$. In HET the activity rate of each node is drawn randomly from a power-law distribution with support (*ϵ*, 1] and the same average value as in HOM. Lower values of the power-law exponent *γ* correspond to a higher contact heterogeneity. The average degree is the same as in HOM. We chose a population of $\bar{V}=306$ individuals, average length of stay *τ* = 10 days, probability of strain injection per incoming individual *p*_*s*_ = 0.079, and recovery rate *μ* = 0.00192 (see Materials and methods). (A) Sample time series of strain abundance for HOM and HET with *γ* = 0.7. Each time series is represented with a different color. All abundances are stacked together, so that plot’s height represents prevalence. Here *β* = 0.02. (B) Average prevalence vs *β*, and (C) average richness vs *β*. Two levels of heterogeneity are here considered for HET. For the sake of visualization, the shaded area corresponding to the standard deviation is shown only for HOM. Median and confidence intervals are reported in S1 Fig of the supporting information. (D) Average prevalence vs richness. Dashed lines are shown as a guide to the eye, highlighting variation in richness induced by network topology.

Fig 1 shows the results of numerical simulations comparing HOM and HET models. Sample epidemic trajectories are reported in Fig 1A. Here every strain is indicated with its own color to display its dynamics resulting from the interaction with the other strains. Fig 1B–1D shows summary statistics in varying strain transmissibility *β*. The prevalence presents a well-known behavior for both static and dynamic networks (Fig 1B): contact heterogeneities lower the transmissibility threshold above which total prevalence is significantly above zero, thus allowing the spread of pathogens with low transmissibility. At the same time, however, heterogeneities hamper the epidemic spread when *β* is large, reducing the equilibrium prevalence [35]. Fig 1C shows the average richness, i.e. the number of distinct strains co-circulating. For low values of *β* HET displays larger richness values compared to HOM. This trend reverses as *β* increases, and the richness is lower in HET consistently with the lower level of prevalence. The relation between richness and prevalence, however, is not straightforward. For instance, the reduction in richness for high *β* values is important even for the case with limited contact heterogeneity, when prevalence is barely affected. The scaling between prevalence and richness is not linear as *β* varies (Fig 1D), and the relation between the two quantities varies appreciably among contact networks. In correspondence of a fixed value of prevalence, heterogeneous networks have lower richness—e.g. a prevalence value of ∼0.8 corresponds to ∼20% lower richness in HET with respect to HOM, as highlighted in Fig 1D.

This fact can be explained by the dynamical properties of epidemics on heterogeneous networks. Active nodes, involved in a larger number of contacts, get infected more frequently [35]. Also, a randomly chosen node is likely surrounded by active nodes [33]. As a consequence, injected strains often find their propagation blocked by active infected nodes. In this way, contact heterogeneities enhance the competition induced by mutual exclusion and hamper the wide-spread of emerging strains, similarly to what was found in [46]. This mechanism is further confirmed by looking at the persistence time of strains (S2 Fig in the supporting information). Above the epidemic threshold, it is on average shorter in heterogeneous networks than in homogeneous ones. The distributions are however more skewed in heterogeneous networks, indicating that more strains are going extinct rapidly, while a few others can survive for a long time in the population.

**Fig 2:**
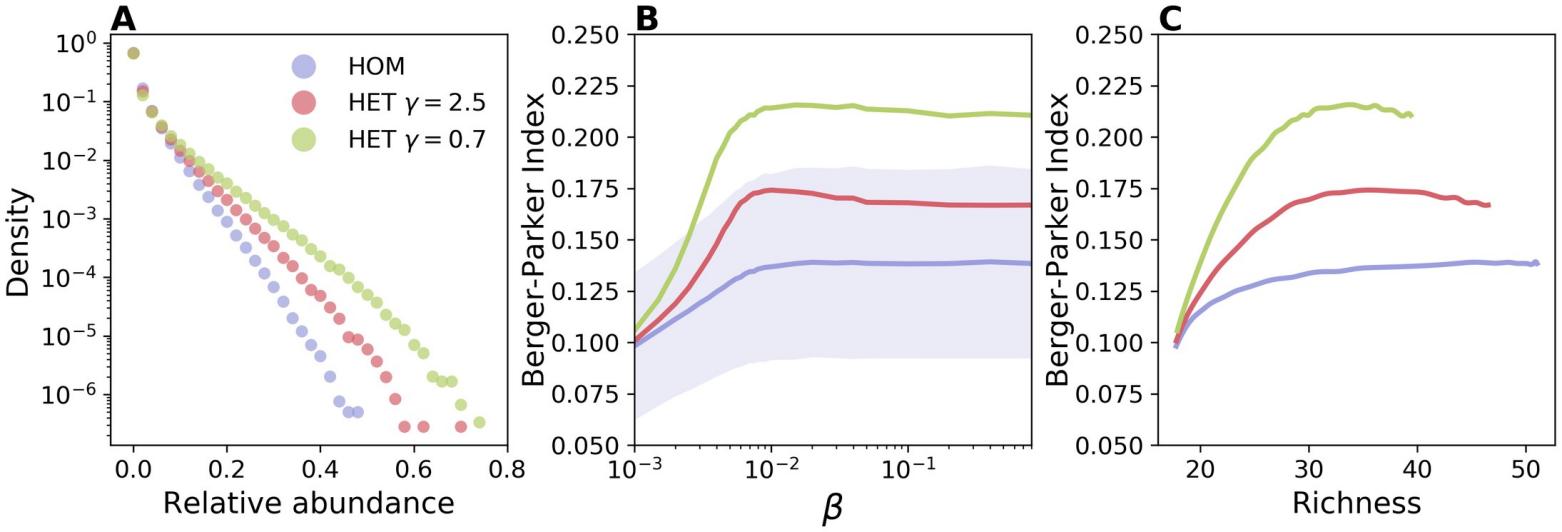
Effect of contact heterogeneity on strain dominance. (A) Distribution of strains’ relative abundance, i.e. the frequency of strains infecting a given fraction of the total prevalence, for HOM (blue), HET with *γ* = 2.5 (red), and HET with *γ* = 0.7 (green). Here *β* = 0.02. (B) Berger-Parker index, i.e. the relative abundance of the most abundant strain, as a function of *β*. For the sake of visualization, the shaded area corresponding to the standard deviation is shown only for HOM. Median and confidence intervals are reported in S1 Fig of the supporting information. (C) Berger-Parker index vs richness. Parameters are the same as in Fig 1.

If on the one hand hubs accelerate the extinction of certain strains, on the other they act as super-spreaders, amplifying the propagation of other strains. We find that this impacts profoundly the distribution of strains’ abundances, i.e. the strain-specific prevalence. Fig 2A shows that the latter is broader for the HET network, with the most abundant strain reaching a larger proportion of cases. This situation is synthesized by the Berger-Parker index, that quantifies the level of unevenness or dominance of a given ecological system [52, 53]. This is defined as the relative abundance of the most abundant strain (see Materials and methods section). Fig 2B shows that Berger-Parker index increases with *β* for all networks. This is expected since at low *β* strains′ transmission chains are short and barely interact, while they interfere more at higher values of transmission potential. The Berger-Parker index is always higher in a heterogeneous network, even when the comparison is made at fixed values of richness (Fig 2C). An alternative indicator, the Shannon evenness, shows a similar behavior as displayed in S3 Fig.

The fraction of strains going extinct also depends on stochastic effects in a finite size population. We indeed found that increasing network size, when temporal and topological properties were the same, led to an increase in both persistence time and richness (S4 Fig). This shows that interference among transmission chains is reduced in larger populations. However, the relative abundance distribution remained similar, showing that it is primarily affected by the nodes’ activity distribution (S5 Fig).

Eventually, we tested whether additional mechanisms of strain injection were leading to different results. In S6 Fig we assumed new strains to infect susceptible nodes already present in the system with rate *q*_*s*_, mimicking in this way transmissions originating from an external source, as it can happen in real cases. The plot of S6 Fig shows the same qualitative behavior described here.

### Effect of community structure

We considered a community model (COM) with *n*_*C*_ communities in which all nodes are as active as in HOM, but direct a fraction *p*_*IN*_ of their links within their community and the rest to nodes in the remaining *n*_*C*_ − 1 communities. The closer *p*_*IN*_ is to 1, the stronger the repartition in communities is.

**Fig 3:**
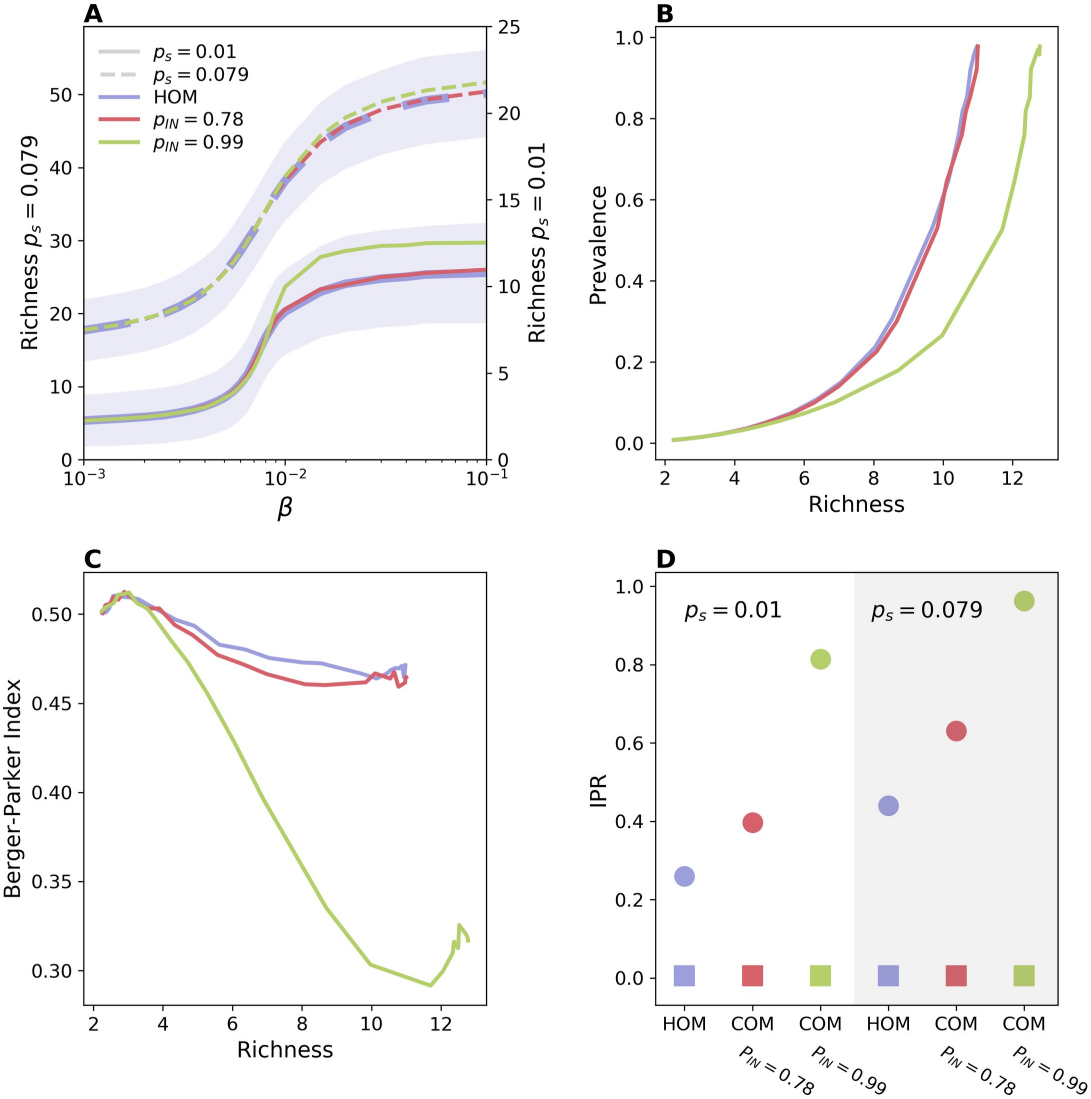
Impact of community structure. (A) Richness vs *β* for HOM (blue), COM with *p*_*IN*_ = 0.78 (red), and COM with *p*_*IN*_ = 0.99 (green). For both COM models we have set *n*_*C*_ = 6. Solid lines correspond to *p*_*s*_ = 0.01, while dashed lines correspond to *p*_*s*_ = 0.079. Solid lines refer to the right y-axis, while dashed ones to the left y-axis. For the sake of visualization, the shaded area corresponding to the standard deviation is shown only for HOM. Median and confidence intervals are reported in S1 Fig of the supporting information. (B) Prevalence vs richness and (C) Berger-Parker index vs richness, for *p*_*s*_ = 0.01. (D) Average *IPR* for both *p*_*s*_ = 0.01 (white background) and *p*_*s*_ = 0.079 (gray background). Here *β* = 0.02. Squares correspond to *IPR* obtained from total prevalence while circles correspond to *IPR* obtained from strains’ abundances. A value of the *IPR* close to 1 indicates localization over one community. Here no injection due to transmission from an external source is assumed (*q*_*s*_ = 0). The effect of this second mechanism is shown in S6 Fig.

Fig 3A and 3B shows that a network with communities displays a higher richness for large *β*; even when community structure barely affects prevalence (Fig 3B). However, the effect is important only when communities are fairly isolated (*p*_*IN*_ = 0.99) and the injection from the outside is not so frequent—otherwise the effect is masked by strain injection which occurs uniformly across communities. In particular, for the values of *p*_*IN*_ = 0.78 and *p*_*s*_ = 0.079, chosen to match the hospital application, the difference with the homogeneous case is very small. The limited role of community structure is also confirmed by the fact that once this feature is combined with heterogeneous activation—in a model with the activation scheme of HET and the stub-matching of COM—the latter property has the dominant effect and the richness decreases (S1 Fig).

The relation between richness and prevalence remains the same when adding the injection of new strains due to the transmission from an external source. This mechanism further increases the richness. When *β* is high and the fraction of infected nodes is close to one, however, such a mechanism is hindered by the fact that susceptible nodes, that can get infected from the external source, are rare (see S6 Fig). This is why richness starts to decrease for high values of *β*.

We tested the consequences of communities in strain dominance by plotting the Berger-Parker index in Fig 3C. For low *β*, the behavior of the Berger-Parker index follows the trend in richness. The initial decrease in this indicator is due to the increase in richness, that occurs at constant prevalence and is thus associated to a decrease in the average abundance [54]—green curve corresponding to *p*_*IN*_ = 0.99 and *p*_*s*_ = 0.01. At larger values of *β*, instead, increased competition levels induced higher dominance levels.

The increase in strain diversity is due to the reduced competition among strains introduced in different communities. When coupling among communities is low, indeed, strains may spend the majority of time within the community they were injected in, thus avoiding strains injected in other communities. Fig 3D confirms this hypothesis by showing the Inverse Participation Ratio (*IPR*) [55] that quantifies uniformity in the repartition of abundance across communities. Values close to zero indicate uniform repartition, while, conversely, values close to 1 indicate that, on average, a strain is confined within a single community for most of the time (more details are reported in the Materials and methods section). The strength of the community structure does not affect the repartition of the total prevalence (squares in the plot), however it alters the average *IPR* value computed from the abundance of single strains, thus strains become more localized as *p*_*IN*_ increases. Notice that a certain degree of localization is present also in the homogeneous network, due to those strains causing very few generations before going extinct.

As a sensitivity analysis we tested whether the main results obtained so far are the same in a more realistic situation where additional heterogeneous properties of nodes are accounted for. We consider the case in which infectious duration varies across individuals, as happens for *S. aureus* colonization. S7 Fig shows that the inclusion of three classes differing in recovery rate reduces richness and increases the Berger-Parker index with respect to the homogeneous recovery. However, the effects discussed so far—e.g. reduction and amplification of richness in HET and COM, respectively—are still present.

### Effect of turnover of individuals

**Fig 4:**
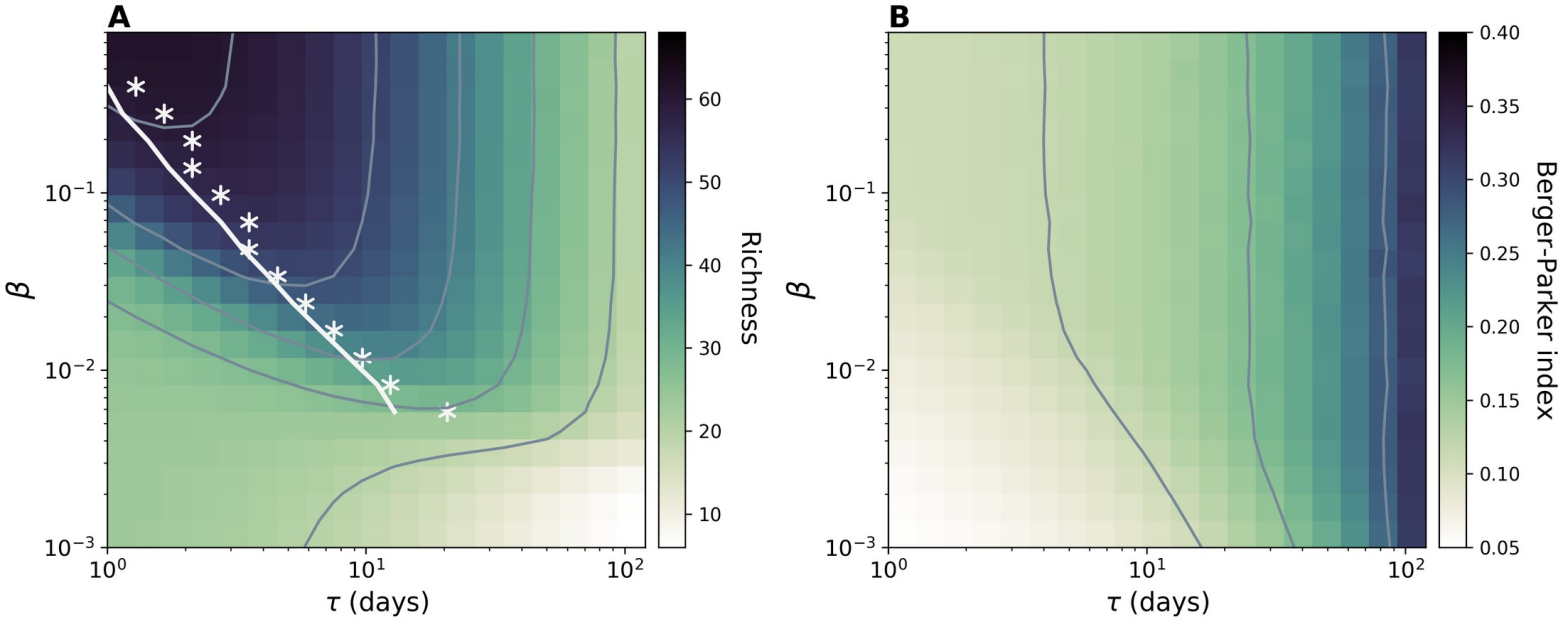
Effects of node length of stay on strain diversity. (A) Average richness and (B) Average Berger-Parker index for simulations on HOM model. Contour plots are shown in both figures. While exploring *τ* we also set the value of the average network size $\bar{V}$ to 306, thus the injection rate can be computed by the relation $\lambda_{in}=\bar{V}/\tau$. For each value of *β* we highlight in panel (A) the value of the length of stay corresponding to the maximum richness (white asterisks) together with the analytical prediction (white line) obtained by using Eq (1). Here *μ* = 0.00192, $\bar{k}=0.89$, *a*_*H*_ = 0.28.

Node turnover represents another important property of a network that may impact the ecological dynamics of strains for two reasons: incoming individuals contribute to richness by injecting new strains; on the other hand, the removal from the population of infected nodes breaks transmission chains and hampers the persistence of strains. The result of the interplay between these two mechanisms is summarized by the plot of richness as a function of *β* and node length of stay, *τ*,—Fig 4A. The figure, obtained with the HOM model, shows two distinct regimes. In the former case, richness decreases as *τ* increases, because replacement of individuals becomes slower and injections less frequent. In the high *β* regime, instead, the average richness at fixed *β* does not depend monotonically on the node turnover but it is instead maximized at intermediate *τ*. Interestingly, the optimal value of *τ* decreases as *β* increases. This behavior can be explained by looking at the balance between injection and extinction that determines the equilibrium value of richness, ${\bar{N}}_{S}$. This reads [56]:

$$\begin{array}{c} {\bar{N}}_{S}=\lambda_{\text{in}}p_{s}\;T_{\text{pers}}\left(\beta ,\tau \right)=\bar{V}\;p_{s}\;\frac{T_{\text{pers}}\left(\beta ,\tau \right)}{\tau }, \end{array}$$ (1)

where λ_in_*p*_*s*_ is the rate at which new strains are introduced and *T*_pers_ is the average persistence time of a strain. The trade-off between injection and extinction appears as the ratio between the two time scales, *T*_pers_ and *τ*. In the limit *τ* → 0 the spread plays no role, even for high *β*. As *τ* increases, newly introduced infectious seeds have a higher probability to spread, thus the average extinction time initially increases super-linearly with *τ* (see S8 Fig in the supporting information) resulting in an increase of richness. However, past a certain value of *τ*, *T*_pers_ does not grow super-linearly anymore, thus a further increase in *τ* is detrimental for pathogen diversity because it is associated to fewer introductions. This general behavior was not altered by the accounting for introductions by transmissions from an external source as shown in S6 Fig.

We derive an approximate formula for *T*_pers_ considering an emerging strain competing with a single effective strain formed by all other strains grouped together. This formulation, enabled by the neutral hypothesis, makes it possible to write the master equation describing the dynamics and to use the Fokker-Planck approximation to derive persistence times (see Materials and methods section). Analytical results well reproduce the behavior observed in the simulations, and, in particular, the value of the length of stay maximizing richness for different *β* as shown by the comparison between white stars and continuous line in Fig 4B. The quantitative match for other values of *p*_*s*_ is reported in S9 Fig.

Unlike richness, Berger-Parker index always increases monotonically with the length of stay—Fig 4B. This behavior is due to the correlation of this indicator with average abundance, similarly to what we discussed in the previous section.

### Spread of *S. aureus* in a hospital setting

We conclude by analyzing the real-case example of the *S. aureus* spread in a hospital setting [10, 57]. We used close-proximity-interaction (CPI) data recorded in a long-term health-care facility during 4 months by the i-Bird study [16, 28, 31]. These describe a high-resolution dynamical network whose complex structure reflects the hospital organization, the subdivision in wards and the admission and discharge of patients [58]. Together with the measurements of contacts, weekly nasal swabs were routinely performed to monitor the *S. aureus* carriage status of the participants and to identify the spa-type and the antibiotic resistance profile of the colonizing strains.

The modeling framework considered here well applies to this case. The SIS model is widely adopted for modeling the *S. aureus* colonization [59, 60], and the assumption of mutual exclusion is made by the majority of works to model the high level of cross-protection recognized by both epidemiological and microbiological studies [61, 62]. The dynamic CPI network was previously shown to be associated with paths of strain propagation [16]. Consistently, we assumed that transmission is mediated by network links with transmissibility *β*. In addition, new strains are introduced in the population carried by incoming patients, or through contacts with persons not taking part in the study.

**Fig 5:**
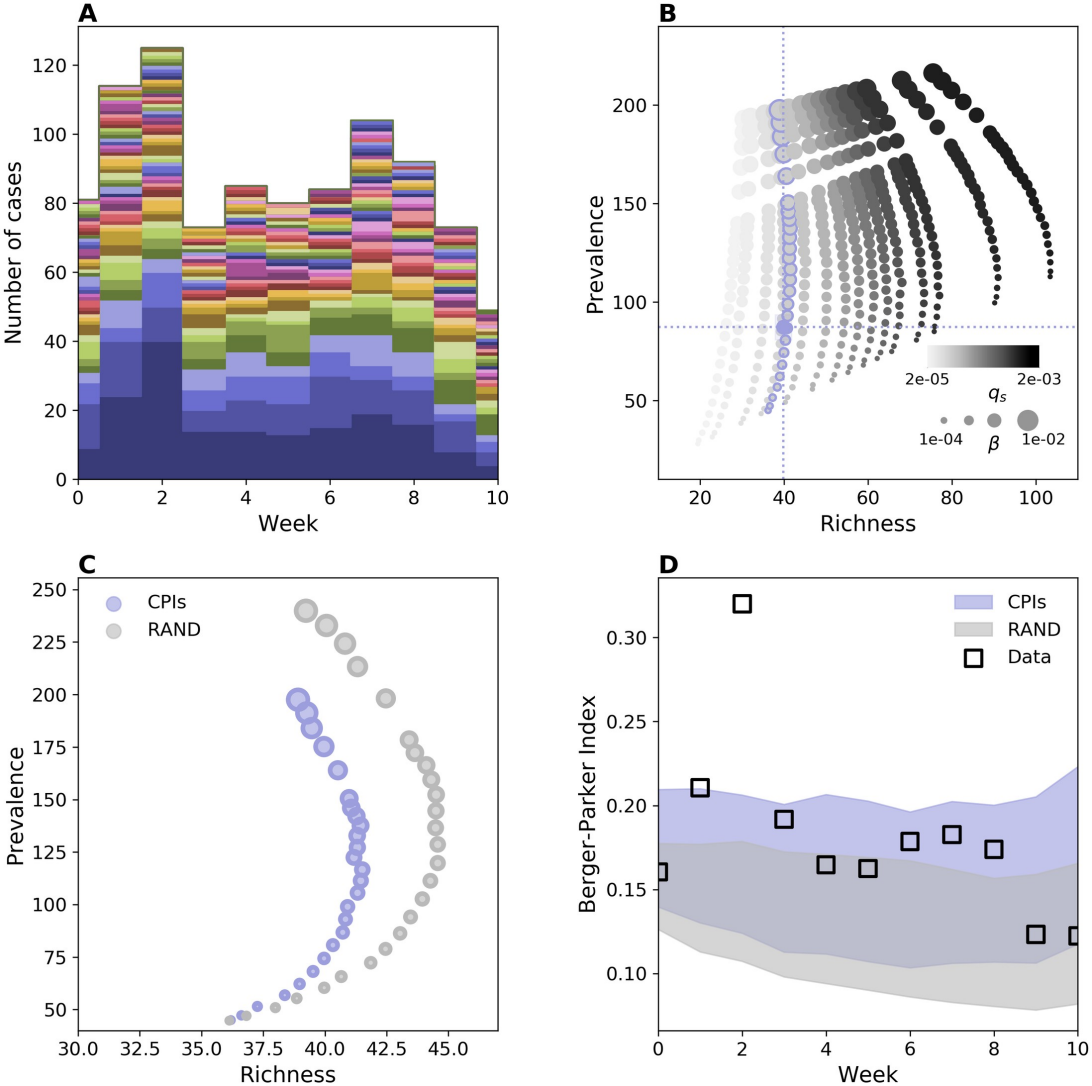
*S. aureus* population structure on hospital network. (A) Weekly carriage data measured during the i-Bird experiment. Each *S. aureus* strain’s abundance time series is represented by a different color. All time series have been stacked as in Fig 1A. (B) Prevalence vs. richness from simulations on the hospital network for different *β* and different rates of introduction, here tuned by the parameter *q*_*s*_. Blue dashed lines represent the average empirical values. (C) Prevalence vs richness for hospital contact data (blue dots) and RAND null model (grey dots). In RAND contacts are randomized by preserving the first and the last contact of every individual. Markers’ size in (B) and (C) is proportional to the value of *β*. The hospital curve corresponds to the curve in (B) with blue-contour markers. (D) Weekly value of Berger-Parker index in carriage data (squares) along with the same quantity from the simulations. The shaded areas indicate the average plus/minus the standard deviation obtained from 1000 stochastic runs. For each network, parameter values are the ones that reproduce empirical prevalence and richness. Duration of colonization is assumed here to be 35 days [63]. Alternative values of this parameter led to the same qualitative results (S10 Fig).

Fig 5A shows weekly carriage and its breakdown in different strains. Prevalence and richness fluctuate around the average values 87,3 ± 6,3 cases and 39,8 ± 2 strains, respectively. Simulation results are reported in Fig 5B, that displays the impact of transmission and introduction rate on richness and prevalence. When *q*_*s*_ is low we find a positive trend between richness and prevalence, consistently with the synthetic case. For larger values of *q*_*s*_ the trend appears instead different. As transmissibility increases, richness initially grows with prevalence and then decreases after a certain point. This behavior is the same as observed in S6 Fig and stems from the reduction of susceptible nodes, that causes a decline in the expected injection rate—see Materials and methods section.

To quantify the effect of contact patterns on *S. aureus* population ecology we compared simulation results with the ones on a network null model. Specifically, we built the RAND null model that randomizes contacts while preserving just the first and the last contact of every individual. The randomization preserves node turnover, the number of active nodes and links and destroys contact heterogeneities and community structure along with other correlations. Fig 5C shows the comparison for different transmissibility values. The effect of the network is consistent with the theoretical results described for a heterogeneous network, i.e. smaller richness values correspond to the same prevalence in the real network compared to the homogeneous one. We then quantified the level of dominance of the multi-strain distribution by means of the Berger-Parker index. We chose for each network the values of *q*_*s*_ and *β* that better reproduce empirical richness and prevalence and, interestingly, we found that, for the two cases, same average richness and prevalence correspond to different levels of Berger-Parker index. The Berger-Parker index obtained with the real network is the highest and the one that better matches the empirical values—i.e. the empirical values are within one standard deviation of the mean for almost all weeks. Based on this result we argue that contact heterogeneities, along with the other properties of the contact network, contribute to the increased dominance of certain strains.

## Discussion

Multiple biological and environmental factors concur in shaping pathogen diversity. We focused here on the host contact network and we used a minimal transmission model to assess the impact of this ingredient on strain population ecology, quantifying the effects of three main network properties, i.e. heterogeneous activity potential, presence of communities and turnover of individuals. Results show that the structure and dynamics of contacts can alter profoundly strains’ co-circulation. Contact heterogeneities were found to shape the distribution of strains’ abundances. Highly active nodes are known to play an important role in outbreak dynamics by acting as super-spreaders [33]. At the same time, however, they were found to enhance the interference between the transmission chains of different strains, thus hindering the spread of an emerging variant [46]. Here we showed that the combination of these two dynamical mechanisms reduces the richness and increases the level of heterogeneity in strains’ abundances. In particular, hubs could allow strains with no biological advantage to generate a large number of cases and outcompete other equally fit strains. This mechanism may potentially bias the interpretation of biological data. Dynamical models that do not properly account for contact structure could overestimate the difference in strains’ epidemiological traits in the attempt to explain observed fluctuations in strain abundance induced in reality by super-spreading events. Moreover, these models could provide biased assessment of transmission vs. introduction rates.

The presence of communities causes the separation of strains and mitigates the effect of competition thus enhancing co-existence. A similar behavior was already pointed out before [46, 51, 59, 64], e.g. for the spread of *S. pneumoniae*, as induced by age assortativity [64], for the case of *S. aureus* where distinct settings were considered [59], and for a population of antigenic distinct strains in presence of cross-immunity [51]. We found that the impact of community structure is not so strong, and it is likely minor when individuals of different communities have frequent contacts. No appreciable variation was observed, indeed, for *p*_IN_ = 0.78, chosen to match the inter-ward coupling of the hospital network. Similar results can be expected for school classes or workplace departments presenting a similar level of community mixing. The effect on richness becomes appreciable for low community coupling (e.g. *p*_IN_ = 0.99 in Fig 3). This is consistent with a certain degree of diversity observed among strains belonging to separated communities, as it is the case of different hospitals [15].

Eventually, the analysis of turnover of individuals revealed major effects on strain diversity, when this mechanism is also the main driver of the introduction of strains in the population. When transmissibility is low richness decreases with host length of stay. When transmissibility is above the epidemic threshold we showed the existence of an optimal value of the length of stay that maximizes strain richness as a result of the interplay between two competing time scales, namely the typical inter-introduction time and the average persistence time of a strain. This provides insights for the spread of bacterial infections in transmission settings, such as hospitals or farms, that are of particular relevance for the spread of antimicrobial resistance and that are characterized by a rapid host turnover [15, 31, 65]. For the case of hospitals, for instance, they suggest that variations in patients’ length of stay, as induced by a change of policy, could have appreciable effects on the population structure of nosocomial pathogens.

We adopted a neutral model to better disentangle the relative role of the different network properties. A wide disease-ecology literature addressed the consequences of neutral hypotheses on multi-strain balance in order to provide a benchmark for interpreting the observed co-existence patterns and gauging the effect of selective forces potentially at play [11, 18, 66, 67]. Many of these works addressed, for instance, the co-existence between susceptible and resistant strains of *S. pneumoniae* [11, 66]. However, this assumption was rarely adopted in network models, that consider for the majority strains with different epidemiological traits with the aim of describing pathogen selection and evolution [47–49, 68]. Strains were assumed to have the same infection parameters in [50, 51], where the role of community structure and clustering was analyzed in conjunction with cross-immunity. With respect to these works, the minimal transmission model used here enabled a transparent comprehension of the role of the network. Multiple identical SIS processes can be mapped, in fact, on a single SIS process, in such a way that the wide literature of single SIS processes allows for a better understanding of the behavior recovered in the simulations [32, 33]. Strains can be also grouped in two macro-strains. This strategy allowed us to adopt the viewpoint of an emerging strain and study its competition with the others seen as a unique macro-strain. The associated master equation and Fokker-Planck approximation allowed computing the average extinction time, capturing the key aspects of the dynamics. In a future work this theoretical framework could be extended to consider other network topologies. It could, for instance, be coupled with the activity-block approximation to describe heterogeneous networks. Additional numerical analyses, based on a similar transmission model, could also address other properties known to alter spreading dynamics, such as heterogeneous inter-contact time distribution or topological and temporal correlations.

As a case study, we analyzed the spread of *S. aureus* in a hospital taking advantage of the simultaneous availability of contact and carriage information [16]. The temporal and topological features of the network lead to a lower prevalence and richness with respect to the homogeneous mixing (although the effect was quite small). In addition, similar prevalence and richness values are associated to different dominance levels in different networks—i.e. different values of the Berger-Parker index—with the real network leading to a higher dominance as observed in reality. This behavior can be explained by the theoretical results and can be attributed essentially to the effect of contact heterogeneities, considering that the community structure does not have appreciable effects for this network, as discussed above. The importance of accounting for host contacts and hospital organization in the assessment of bacterial spread and designing interventions has been recognized by several studies [16, 28–31, 63]. Here we show that this element may be critical also for understanding the population ecology of the bacterium. It is important to note however that, while the realistic network provides results that are closer to the data, this ingredient explains only part of the heterogeneity observed in the abundance. This shows that the contact network is a relevant factor, but other factors should be considered as well. The approach used here is intentionally simplified, as we focused on the main dynamical consequences of the contact network. Clearly, more detailed models can be designed to reproduce more closely the data. A certain degree of variation in the epidemiological traits could be at play, as for example the fitness cost of resistance [8]. Role of hosts in the network (e.g. patients vs. health-care workers), and heterogeneities in health conditions, antibiotic treatment and hygiene practices are also known to affect duration of carriage and chance of transmission [16, 28, 31, 63]. Eventually, we must consider that the comparison of model output with carriage data is also affected by the limitation of the dataset itself, already described in [16]. In particular, the weekly swabs may leave transient colonization undetected. Moreover, while the relevance of CPIs as proxies for epidemiological links has been demonstrated [16], the transmission through the environment (e.g. in the form of fomites) is also possible.

The understanding provided here can be relevant for other population settings, temporal scales and geographical levels. In addition, the modeling framework could be applied to pathogens other than *S. aureus*, such as *human papillomavirus*, *S. pneumoniae* and *Neisseria meningitidis*, for which the strong interest in the study of the strain ecology is justified by the public health need for understanding and anticipating trends in antibiotic resistance, or the long-term effect of vaccination [1, 2, 4, 5]. With this respect, if the simple framework introduced here increases our theoretical comprehension of the multi-strain dynamics, more tailored models may become necessary according to the specific case. In particular, we have considered complete mutual exclusion as the only mechanism for competition. In reality, a secondary inoculation in a host that is already a carrier may give rise to alternative outcomes, such as co-infection or replacement [69]. In addition, infection or carriage may confer a certain level of long-lasting strain-specific protection and/or a short-duration transcendent immunity [11, 50]. Eventually mechanisms of mutation and/or recombination are at play and their inclusion into the model can be important according to the time scale of interest.

## Materials and methods

### Network models

We provide here details of the generative algorithms used for the contact network models. Network dynamics is implemented in discrete time according to the following rules common to all models:

**Turnover dynamics**: new nodes arrive according to a Poisson process with rate λ_in_ and leave after a random time drawn from an exponential probability distribution with average *τ*. After a short initial transient, population size is Poisson distributed with average $\bar{V}=\lambda_{\text{in}}\tau$. Upon admission, a node *i* is assigned with an activity potential *a*_*i*_, i.e. an activation rate, drawn at random from a given probability distribution *P*(*a*). Any node retains this property throughout its whole lifespan.

**Activation Pattern**: each node *i* becomes active with rate *a*_*i*_. It then receives a number of stubs drawn from a zero-truncated Poisson distribution with parameter *κ*—we require active nodes to engage in at least one contact. The average number of stubs, computed among active nodes, is thus given by *κ*/(1 − *e*^−*κ*^), and the average degree can be computed by the latter quantity multiplied by the average activity potential. The active status lasts for a single time step.

**Stub-matching**: stubs are then matched according to the actual model considered.

We now describe in detail each network model:

**HOM**: in this model each node has the same probability *a*_H_ to be active during each time step; the activity distribution is thus *P*(*a*) = *δ*(*a* − *a*_H_), where *δ*(*x*) is the Dirac’s delta function. Stubs are matched completely at random in order to form links, according to a configuration model [33]. We discard eventual self-links and multiple links that may occur during the matching procedure.

**HET**: here each node *i* has its own activity rate *a*_*i*_, drawn from a power-law distribution *P*(*a*) ∝ *a*^−*γ*^, with *a* ∈ (*ϵ*, 1]. We tune the variance by varying *γ*—lower *γ* higher variance. We then set *ϵ* to have the average activity $\bar{a}$ equal to *a*_H_ in HOM. Stub-matching procedure is the same as in HOM. HET model is thus a variant of the activity driven model introduced in [34] with the difference that here contacts are created only among active individuals.

**COM**: incoming nodes are assigned to one among *n*_*C*_ communities with equal probability—so that communities have the same size on average—and belong to the same community throughout their whole lifespan. Stubs are matched according to the community each node belongs to. Precisely, any stub is matched either with another stub of the same community, with probability *p*_*IN*_, or with a stub of a different community, with complementary probability. Here the stub-matching procedure results in a larger number of lost links—to eliminate multiple links and self-loops—compared to HOM and HET, due to the difficulty in matching stubs within small groups. Thus, the parameter *κ* has to be adjusted manually to recover the same average degree as in HOM and HET. Each node has the same activity potential *a*_H_ as in HOM.

### Hospital network and null model

We use a dynamical contact network obtained from CPI data collected during the i-Bird study in a French hospital. Details of the network are already reported in [16]. Briefly, the dataset describes contacts occurring between 592 individuals from July to November 2009. The study involved both patients and health-care workers, distributed in 5 wards, as well as hospital service staff. Every participant wore a wireless device designed to broadcast a signal every 30 s containing information about its ID. Signal strength was tuned so that only devices within a small distance (around 1.5 m) were able to register a contact. CPIs were finally aggregated daily, keeping the information about their cumulative duration within each day.

We discard CPIs relative to the first 2 weeks and the last 4 weeks of dataset, corresponding to a period of adjustments in the measurements and progressive dismissal of the experiment, respectively. Simulations conducted with the CPIs network were compared with results obtained with a null model which we refer to as RAND. According to this randomization scheme the activity of a node is randomized while respecting the constraint that removal and addition of contacts must not alter the time of the first and the last contact of each node (*t*_*S*_ and *t*_*L*_ respectively). Notice that RAND preserves the number of nodes that are present at any time in the network by preserving their first contact *t*_*S*_ and their length of stay *t*_*L*_ − *t*_*S*_. Null models randomizing the latter properties lead to misleading results when node length of stay is heterogeneous and node turnover occurs [70]. RAND also sets all contact weights equal to the average weight value.

### Spreading simulations

Spreading dynamics is stochastic and is performed in discrete time. At each time step of duration Δ*t*, we update the state of each node: each infected node transmits the strain it is carrying to a susceptible neighbor with probability *β*Δ*t* and it turns susceptible with probability *μ*Δ*t*. Notice that due to mutual exclusion, an individual can be infected by a single strain at a time [71]. Strain injection is given by the combination of two processes: incoming individuals bring a new strain with probability *p*_*s*_, and susceptible individuals turn infectious with a new strain with probability *q*_*s*_Δ*t*. The two mechanisms mimic respectively incoming infectious individuals (e.g. admission of colonized patients) and transmission from an external source (in the hospital example this corresponds to contacts with individuals that were not participating in the study). The expected injection rate, which accounts for both introduction mechanisms, is thus given by $\iota =\lambda_{\text{in}}p_{s}+\bar{S}q_{s}$, where $\bar{S}$ is the average number of susceptible individuals at the equilibrium. In the theoretical analysis in the main paper we assumed *q*_*s*_ = 0 for simplicity, thus variations in *ι* were induced by variations in λ_in_ and *p*_*s*_. The case *q*_*s*_ > 0 was considered in the supporting information.

Simulations on synthetic networks differ from those on the hospital network in the combination of the spreading and network dynamics. In the synthetic network case, at each time step of duration Δ*t* = 1h, both network and spreading dynamics are simulated one after the other. On average, λ_*in*_Δ*t* new nodes enter in the population per time step, while existing nodes can leave with probability Δ*t*/*τ*. Nodes then form contacts according to the specific generative network algorithm. Eventually, transmission and recovery are simulated as explained above. In order to reconstruct the equilibrium dynamics we run simulations for a sufficiently long time span, discarding a transient time of 4 ⋅ 10^4^ time steps. We verified that the dynamical properties at the equilibrium are unaffected by initial conditions.

For the hospital example, the network is an external parameter fed into the simulations. Contacts were aggregated daily keeping the information of their total duration. We used this information by considering a weighted network with the link weight, *w*_*ij*_, representing the number of contacts of duration 30 s registered during the day between *i* and *j*. We then assumed Δ*t* = 1 day and computed the probability of infection depending on the weight as $1-(1-\beta \delta {)}^{w_{ij}}$, with *δ* = 30 s. We initialized the system with the same configuration observed in the data, i.e. the initial status for each node is set according to *S. aureus* carriage during the starting week. Simulation length is bound to the hospital contact network duration.

In order to facilitate the comparison between the synthetic and the real scenarios, parameters of the network models were set based on the properties of the hospital network. The average size, the average activity potential and the average degree were set equal to the values estimated from the hospital network, i.e. $\bar{V}=306$, $\bar{a}=0.28$, $\bar{k}=0.89$ respectively. For the COM model the number of communities (*n*_*C*_ = 6) and one of the two explored values of *p*_*IN*_ (*p*_*IN*_ = 0.78) were also informed by the data. Additional values of $\bar{V}$ and *p*_*IN*_ were also tested. Epidemiological parameters were informed by the data in some cases—*p*_*s*_ = 0.079 as computed from carriage data -, or chosen among plausible values for the *S. aureus* colonization—i.e. *μ*^−1^, that was set equal to either 21 or 35 days with other values from 14 to 49 days explored in the supporting information. Values of *β* were explored systematically. For consistency, values of rates throughout the manuscript were always expressed per hour.

### Analysis of carriage data

Carriage data was obtained from weekly swabs in multiple body areas, including the nares. Swabs that resulted positive to *S. aureus* were further examined. Spa-type and antibiotic resistance profiles (MSSA or MRSA) were then determined. In this work we regard two strains as different if they differ in spa-type and/or antibiotic resistance profile. We considered carriage data obtained from nasal swabs dismissing other body areas since the anterior nares represent the most important niche for *S. aureus* [72].

### Ecological measures and other indicators

We described strain population diversity through standard ecological indicators. The abundance of a strain *i*, *N*_*i*_, is the strain-associated prevalence. From this quantity we computed the relative abundance, $f_{i}=\frac{N_{i}}{\sum_{i}N_{i}}$, and the relative abundance distribution, being the frequency of strains with relative abundance *f*. The Berger-Parker index is the relative abundance of the dominant strain, i.e. max_*i*_ *f*_*i*_.

To analyze repartition of strains across communities we use the Inverse Participation Ratio (*IPR*) [55]. The general definition of this quantity is the following. Given a vector $\vec{v}$ with *l* components {*v*_*i*_}_*i*=1,…,*l*_, all within the range [0, 1], the *IPR* is given by:

$$\begin{array}{c} IPR=\sum_{i=1}^{l}v_{i}^{4}. \end{array}$$ (2)

If all the components are of the order (*l*^−1^) then the *IPR* is small. Instead if one component *v*_*i*_ ∼ 1 then *IPR* ∼ 1 too, reflecting localization of $\vec{v}$. The IPR for total prevalence is computed by setting *v*_*i*_ equal to the fraction of infected individuals belonging to community *i* = 1, …, *l* = *n*_*C*_, while the *IPR* for a single strain is computed by setting *v*_*i*_ equal to the fraction of individuals infected by that particular strain and belonging to community *i*. We can extend the *IPR* computation to HOM case by assigning nodes to different groups as in COM but without affecting the stub-matching scheme.

### Analytical results for the homogeneous network

In order to estimate the value of the length of stay maximizing the average richness for a given value of *β* when the contact structure is given by the HOM network we consider a homogeneous mixing version of our system.

Due to Eq (1) the calculation of the average richness reduces to the calculation of the average persistence time. In order to estimate such quantity we focus on a particular strain, labelled as “strain A”, which is injected at *t* = 0 and we group all other strains under the label “strain B”. We are allowed to do so because all strains have identical parameters. We therefore reduce our initial, multi-strain problem, to a two-strain problem. Since all new strains that will be injected after *t* = 0 will be labeled as strain B, it is clear that A is doomed to extinction since there exists an infinite reservoir of B. The average time to extinction is therefore the average time to extinction of strain A.

Since HOM network realizes quite well homogeneous mixing conditions we regard our system as homogeneously mixed. Within this framework it is sufficient to specify the numbers of hosts infected by strain A (*n*_*A*_), hosts infected by strain B (*n*_*B*_) and susceptible hosts (*n*_*s*_). The master equation for the joint probability distribution *P*(*n*_*A*_, *n*_*B*_, *n*_*s*_) is given by [73]:

$$\begin{array}{cc} \dot{P}\left(n_{A},n_{B},n_{s}\right) & =\beta^{\prime }{\bar{V}}^{-1}\left(n_{A}-1\right)\left(n_{s}+1\right)P\left(n_{A}-1,n_{B},n_{s}+1\right)\\ & +\beta^{\prime }{\bar{V}}^{-1}\left(n_{B}-1\right)\left(n_{s}+1\right)P\left(n_{A},n_{B}-1,n_{s}+1\right)\\ & +\mu \left(n_{A}+1\right)P\left(n_{A}+1,n_{B},n_{s}-1\right)+\mu \left(n_{B}+1\right)P\left(n_{A},n_{B}+1,n_{s}-1\right)\\ & +\lambda_{out}\left(n_{A}+1\right)P\left(n_{A}+1,n_{B},n_{s}\right)\\ & +\lambda_{out}\left(n_{B}+1\right)P\left(n_{A},n_{B}+1,n_{s}\right)+\lambda_{out}\left(n_{s}+1\right)P\left(n_{A},n_{B},n_{s}+1\right)\\ & +\lambda_{out}\bar{V}p_{s}P\left(n_{A},n_{B}-1,n_{s}\right)+\lambda_{out}\bar{V}\left(1-p_{s}\right)P\left(n_{A},n_{B},n_{s}-1\right)\\ & -[\left(n_{A}+n_{B}\right)\left(\beta^{\prime }{\bar{V}}^{-1}n_{s}+\mu \right)+\lambda_{out}\left(n_{A}+n_{B}+n_{s}\right)+\lambda_{out}\bar{V}]P\left(n_{A},n_{B},n_{s}\right), \end{array}$$ (3)

Where $\beta^{\prime }=\beta \bar{k}$. Terms appearing on the right-hand side of the equation represent the probability flow associated to each transition event. The first four terms describe, in order, the infection due to strain A, the infection due to strain B, the recovery from A and the recovery from B. The remaining terms are then associated to the discharge of either one of the three types of individuals—infected with A, infected with B and susceptibles—and to the admission of infected of type B and susceptibles respectively. In order to obtain some approximate solution to this equation we assume that the average number of individuals *n*_*A*_ + *n*_*B*_ + *n*_*s*_ and the total prevalence *n*_*A*_ + *n*_*B*_ do not fluctuate in time and are therefore equal to $\bar{V}$ and $i\left(\infty \right)\bar{V}$ respectively, where *i*(∞) is given by:

$$\begin{array}{c} i\left(\infty \right)=\frac{\beta^{\prime }-\mu -\lambda_{out}+\sqrt{{\left(\beta^{\prime }-\mu -\lambda_{out}\right)}^{2}+4\beta^{\prime }\lambda_{out}p_{s}}}{2\beta^{\prime }}. \end{array}$$ (4)

After performing the Van-Kampen size expansion we are left with a Fokker-Planck equation for the density of A $f\left(x=\frac{n_{A}}{\bar{V}}\right)=P\left(n_{A}\right)$:

$$\begin{array}{c} \partial_{t}f=-\partial_{x}(D_{1}\left(x\right)f)+\frac{1}{2\bar{V}}\partial_{x}^{2}(D_{2}\left(x\right)f), \end{array}$$ (5)

where *D*_1_ = *β*′ (1 − *i*(∞)) *x* − *μ* − λ_*out*_ and *D*_2_ = *β*′ (1 − *i*(∞)) *x* + *μ* + λ_*out*_ are the so-called drift and diffusion coefficients respectively.

According to the theory of stochastic processes [73] the average extinction time *T*_pers_(*x*_0_) (where *x*_0_ represents the initial density of strain A) satisfies:

$$\begin{array}{c} D_{1}\left(x_{0}\right)\frac{d}{dx_{0}}T_{\text{pers}}+\frac{1}{2\bar{V}}D_{2}\left(x_{0}\right)\frac{d^{2}}{dx_{0}^{2}}T_{\text{pers}}=-1, \end{array}$$ (6)

with boundary conditions *T*_pers_(0) = 0 and $\frac{d}{dx_{0}}T_{\text{pers}}\left(i\left(\infty \right)\right)=0$. The solution is finally given by:

$$\begin{array}{c} T_{\text{pers}}\left(x_{0}\right)=\frac{i(\infty )}{\lambda_{out}p_{s}}[Ei\left(-\alpha i\left(\infty \right)\right)\left(e^{\alpha x_{0}}-1\right)-e^{\alpha x_{0}}Ei\left(-\alpha x_{0}\right)+ln\left(\alpha x_{0}\right)+\gamma_{E}], \end{array}$$ (7)

where *Ei*(*x*) is the exponential integral function and *γ*_*E*_ is Euler-Mascheroni constant. When a new strain is introduced its prevalence is just 1, therefore we estimate the average extinction time using $T_{\text{pers}}\left(x_{0}={\bar{V}}^{-1}\right)$.

## Supporting information

S1 Text Multi-strain model with heterogeneous recovery classes. This file contains additional information about simulations with individuals grouped into classes with different recovery rates.
(PDF)

S1 Fig Richness and Berger-Parker index as a function of transmissibility for HOM, HET, COM and COM+HET models. Each model is displayed on a different column. HET is characterized by activity distribution exponent *γ* = 0.7. COM+HET model is simulated using the same activation pattern as in HET with *γ* = 0.7 and the same stub-matching procedure as in COM. We consider the case *p*_*IN*_ = 0.99. The first two rows correspond to *p*_*s*_ = 0.01, whereas the last two to *p*_*s*_ = 0.079. For each scenario we show the median (solid line), as well as 50% and 95% CI (shaded areas).
(PNG)

S2 Fig Summary indicators of the persistence time distribution as a function of transmissibility for both HOM and HET models. HOM and HET are displayed in blue and green respectively. (A), (B) and (C) display distribution’s average, coefficient of variation and skewness, respectively. Other parameters are as in Fig 2 in the main text.
(PNG)

S3 FigShannon evenness for HOM model and two instances of HET model.HOM is depicted in blue, whereas instances of HET model with activity distribution exponent *γ* = 2.5 and *γ* = 0.7 are depicted in orange and green respectively. Shaded blue area represents standard deviation for HOM. We introduce the relative abundance of the *i*-th strain: $n_{i}=N_{i}/\sum_{i}N_{i}$, with *N*_*i*_ the abundance of the strain *i* (i.e. the number of infected with strain *i*). Shannon evenness is defined as the normalized Shannon entropy $S\left(\left\{n_{i}\right\}\right)=-N^{-1}\sum_{i}n_{i}\ln n_{i}$, with $N=\ln N_{S}$. Parameters are the same as in Fig 1 in the main text.(PNG)

S4 FigImpact of network size.Richness (A,B), average persistence time (C,D), prevalence (E,F) and Berger-Parker index (G,H) as a function of transmissibility for both HOM and HET models (first and second columns respectively). For each value of $\bar{V}$ we compute *p*_*s*_ to have the strain injection rate, $\bar{V}p_{s}$, the same across the different networks. Other parameters are as in Fig 2 of the main paper. Increasing network size results in a larger number of co-circulating strains, while the re-scaled prevalence and the Berger-Parker index are almost independent of $\bar{V}$. Notice that increasing network size does not lead to any qualitative change in the relation between HOM and HET.(PNG)

S5 FigRelative abundance distribution in varying network size for HOM and HET models.HOM and HET are depicted in blue and green respectively. For each value of $\bar{V}$ we compute *p*_*s*_ to have the strain injection rate, $\bar{V}p_{s}$, the same across the different networks. Other parameters are as in Fig 2 of the main paper.(PNG)

S6 Fig Richness for the different network models with transmission from an external source. The frequency of transmissions from an external source is tuned by *q*_*s*_, which we set here to 0.0002. (A) Richness for HOM model (blue markers) and HET model with activity distribution exponent *γ* = 0.7 (green markers). Here *p*_*s*_ = 0.079. (B) Richness index for HOM model (blue markers) and COM model with within-community connection probability *p*_*IN*_ = 0.99 (green markers). Here *p*_*s*_ = 0.01. (C) Richness as a function of *β* and *τ* for HOM model. Here *p*_*s*_ = 0.079.
(PNG)

S7 Fig Multi-strain dynamics when recovery rate is heterogeneous across individuals. Here, each node belongs to one out of three classes according to its recovery rate—see description in the dedicated section of this supporting information. We compare HOM (blue), HET (green), COM (red) models with and without heterogeneity in the recovery rate (triangles and circles respectively). Panels show prevalence (A), richness (B) and Berger-Parker index (C). Other parameters are like in Figs 1, 2 and 3 in the main paper (*γ* = 0.7 for HET and *p*_*IN*_ = 0.99 for COM).
(PNG)

S8 Fig Average persistence time for HOM in varying transmissibility and length of stay. The quantity is computed from the simulations. The dashed gray line represents a linear trend as a guide to the eye. Parameters are the same as in Fig 4 in the main text.
(PNG)

S9 Fig Comparison between simulations for HOM model and analytical predictions obtained using the Fokker-Planck framework. Solid lines represent average richness obtained by using Eqs (1) and (7) from the main text while dots represent simulations results. Here *β* = 0.04 while other parameters are the same as in Fig 4 in the main text.
(PNG)

S10 Fig Prevalence vs richness for several values of the infectious period and using the CPI network. The value of *q*_*s*_ is the same for the curve highlighted in Fig 5B in the main text, *q*_*s*_ = 0.00018. Here dot size is proportional to the magnitude of *β*.
(PNG)
