# Supplementary material for: Host contact dynamics shapes richness and dominance of pathogen strains
Source: PLoS Comput Biol. 2019 May 21;15(5):e1006530. doi: 10.1371/journal.pcbi.1006530 (PMC6546247; doi:10.1371/journal.pcbi.1006530)
Supplement: S1 Text — This file contains additional information about simulations with individuals grouped into classes with different recovery rates. (PDF) [file pcbi.1006530.s001.pdf]

Supporting Information
Host contact dynamics shapes richness and dominance of pathogen strains

Francesco Pinotti[1], Éric Fleury[2], Didier Guillemot[3], Pierre-Yves Böelle[1], Chiara Poletto[1]

1 Sorbonne Université, INSERM, Institut Pierre Louis d'Épidémiologie et de Santé Publique,Paris,France
2 INRIA, Paris, France
3 Inserm, UVSQ, Institut Pasteur, Université Paris-Saclay, Biostatistics, Biomathematics, Pharmacoepidemiology and Infectious Diseases (B2PHI), Paris, France.

Multi-strain model with heterogeneous recovery classes

In order to test the robustness of our results to the case in which more detailed and realistic ingredients are accounted for, we consider the case in which the recovery rate is heterogeneous across individuals. This mimics a relevant aspect of bacterial colonization, as in e.g. *S. aureus* case. In [1] individuals were subdivided in three main classes with very different carriage duration, i.e. non-carriers, intermittent carriers and persistence carriers. Similarly, we assumed three classes of individuals characterized by different recovery probability per time step, i.e. $\mu^{(1)} = 1$, $\mu^{(2)} = 1.3 \cdot 10^{-3}$ and $\mu^{(3)} = 6.9 \cdot 10^{-4}$. Nodes are distributed in the three classes with probability $p^{(1)} = 0.5$, $p^{(2)} = 0.3$ and $p^{(3)} = 0.2$ [1]. Notice that individuals in the first class recover immediately after a single time step. Recovery rates are chosen so that the average carriage period matches the value used in the main paper.

References

[1] Qiuzhi Chang, Marc Lipsitch, and William P. Hanage. Impact of Host Heterogeneity on the Efficacy of Interventions to Reduce Staphylococcus

aureus Carriage. *Infect Control Hosp Epidemiol*, 37(2):197–204, February 2016.
