# Supplementary figures and images for: Host contact dynamics shapes richness and dominance of pathogen strains
Source: PLoS Comput Biol. 2019 May 21;15(5):e1006530. doi: 10.1371/journal.pcbi.1006530 (PMC6546247; doi:10.1371/journal.pcbi.1006530)

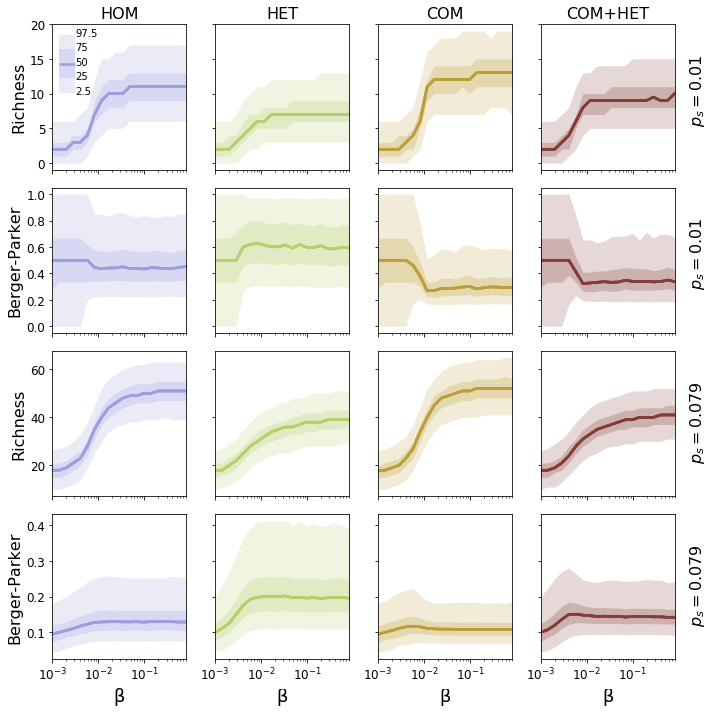

Supplement: S1 Fig — Each model is displayed on a different column. HET is characterized by activity distribution exponent γ = 0.7. COM+HET model is simulated using the same activation pattern as in HET with γ = 0.7 and the same stub-matching procedure as in COM. We consider the case pIN = 0.99. The first two rows correspond to ps = 0.01, whereas the last two to ps = 0.079. For each scenario we show the median (solid line), as well as 50% and 95% CI (shaded areas). (PNG) [file pcbi.1006530.s002.png]

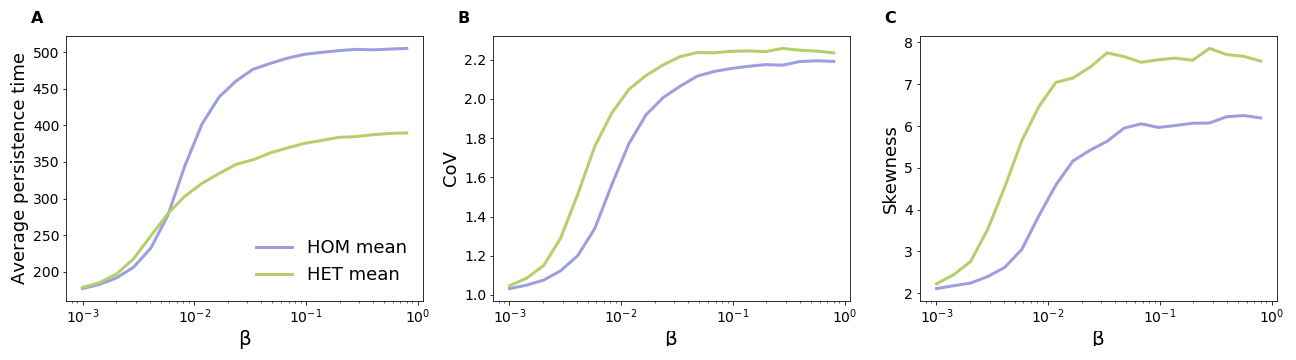

Supplement: S2 Fig — HOM and HET are displayed in blue and green respectively. (A), (B) and (C) display distribution’s average, coefficient of variation and skewness, respectively. Other parameters are as in Fig 2 in the main text. (PNG) [file pcbi.1006530.s003.png]

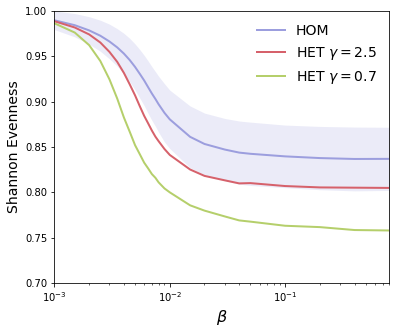

Supplement: S3 Fig — HOM is depicted in blue, whereas instances of HET model with activity distribution exponent γ = 2.5 and γ = 0.7 are depicted in orange and green respectively. Shaded blue area represents standard deviation for HOM. We introduce the relative abundance of the i-th strain: ni=Ni/∑iNi, with Ni the abundance of the strain i (i.e. the number of infected with strain i). Shannon evenness is defined as the normalized Shannon entropy S({ni})=−N−1∑inilnni, with N=lnNS. Parameters are the same as in Fig 1 in the main text. (PNG) [file pcbi.1006530.s004.png]

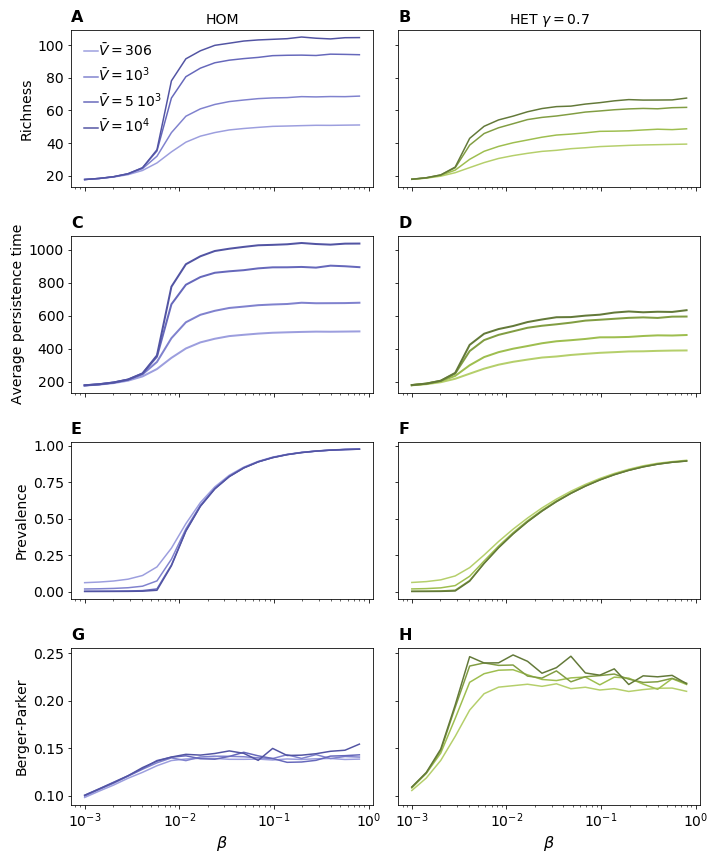

Supplement: S4 Fig — Richness (A,B), average persistence time (C,D), prevalence (E,F) and Berger-Parker index (G,H) as a function of transmissibility for both HOM and HET models (first and second columns respectively). For each value of V¯ we compute ps to have the strain injection rate, V¯ps, the same across the different networks. Other parameters are as in Fig 2 of the main paper. Increasing network size results in a larger number of co-circulating strains, while the re-scaled prevalence and the Berger-Parker index are almost independent of V¯. Notice that increasing network size does not lead to any qualitative change in the relation between HOM and HET. (PNG) [file pcbi.1006530.s005.png]

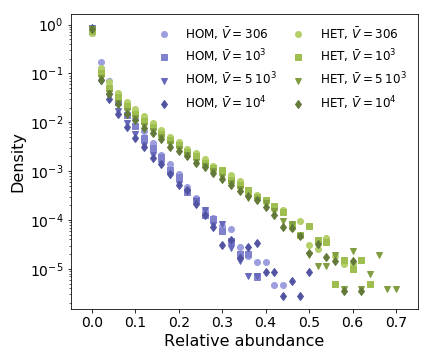

Supplement: S5 Fig — HOM and HET are depicted in blue and green respectively. For each value of V¯ we compute ps to have the strain injection rate, V¯ps, the same across the different networks. Other parameters are as in Fig 2 of the main paper. (PNG) [file pcbi.1006530.s006.png]

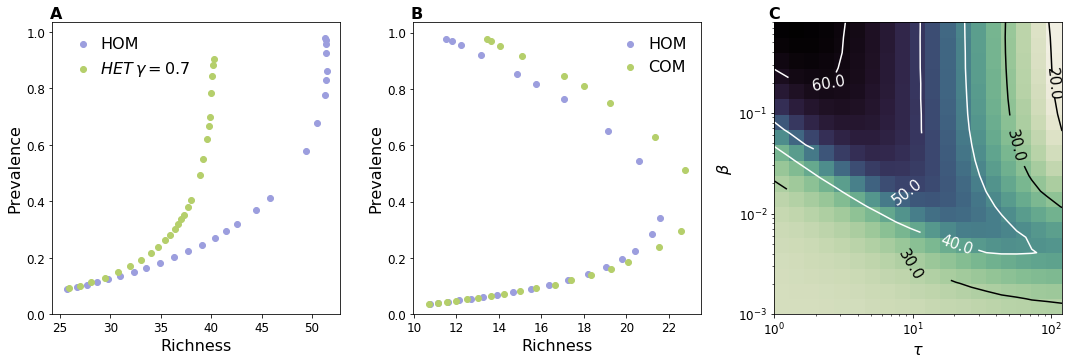

Supplement: S6 Fig — The frequency of transmissions from an external source is tuned by qs, which we set here to 0.0002. (A) Richness for HOM model (blue markers) and HET model with activity distribution exponent γ = 0.7 (green markers). Here ps = 0.079. (B) Richness index for HOM model (blue markers) and COM model with within-community connection probability pIN = 0.99 (green markers). Here ps = 0.01. (C) Richness as a function of β and τ for HOM model. Here ps = 0.079. (PNG) [file pcbi.1006530.s007.png]

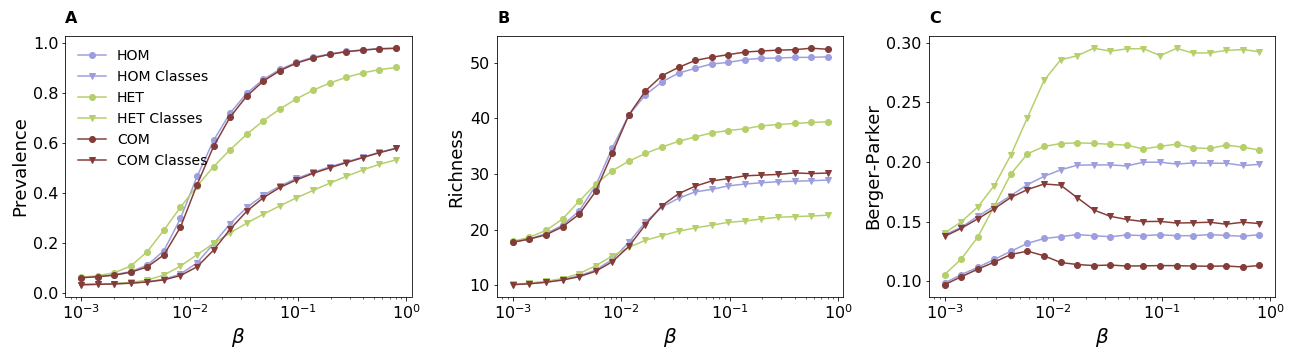

Supplement: S7 Fig — Here, each node belongs to one out of three classes according to its recovery rate—see description in the dedicated section of this supporting information. We compare HOM (blue), HET (green), COM (red) models with and without heterogeneity in the recovery rate (triangles and circles respectively). Panels show prevalence (A), richness (B) and Berger-Parker index (C). Other parameters are like in Figs 1, 2 and 3 in the main paper (γ = 0.7 for HET and pIN = 0.99 for COM). (PNG) [file pcbi.1006530.s008.png]

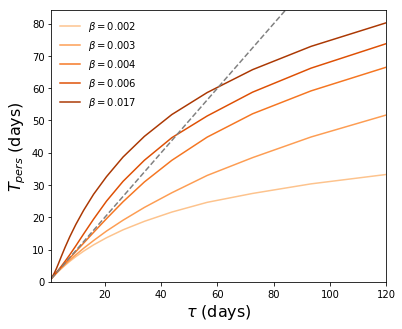

Supplement: S8 Fig — The quantity is computed from the simulations. The dashed gray line represents a linear trend as a guide to the eye. Parameters are the same as in Fig 4 in the main text. (PNG) [file pcbi.1006530.s009.png]

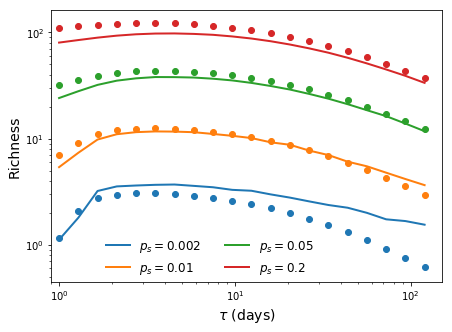

Supplement: S9 Fig — Solid lines represent average richness obtained by using Eqs (1) and (7) from the main text while dots represent simulations results. Here β = 0.04 while other parameters are the same as in Fig 4 in the main text. (PNG) [file pcbi.1006530.s010.png]

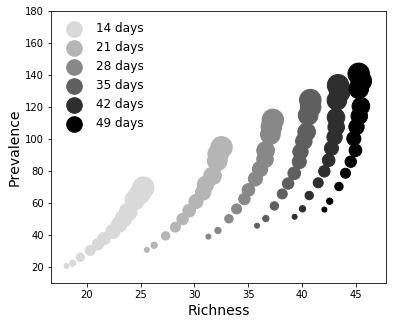

Supplement: S10 Fig — The value of qs is the same for the curve highlighted in Fig 5B in the main text, qs = 0.00018. Here dot size is proportional to the magnitude of β. (PNG) [file pcbi.1006530.s011.png]
